# New species and records of *Venturia* Schrottky (Hymenoptera, Ichneumonidae, Campopleginae) from China and Nepal

**DOI:** 10.3897/zookeys.1041.64238

**Published:** 2021-06-01

**Authors:** Yuan-Yuan Han, Kees van Achterberg, Xue-Xin Chen

**Affiliations:** 1 State Key Lab of Rice Biology, Zhejiang University, Hangzhou 310058, China Zhejiang University Hangzhou China; 2 Ministry of Agriculture Key Lab of Molecular Biology of Crop Pathogens and Insects, Zhejiang University, Hangzhou 310058, China Zhejiang University Hangzhou China; 3 Zhejiang Provincial Key Laboratory of Biology of Crop Pathogens and Insects, Zhejiang University, Hangzhou 310058, China Zhejiang University Hangzhou China; 4 Institute of Insect Sciences, College of Agriculture and Biotechnology, Zhejiang University, Hangzhou 310058, China Zhejiang University Hangzhou China

**Keywords:** Lepidoptera, Oriental region, parasitoid, Pyralidae

## Abstract

Four new species of *Venturia* Schrottky, 1902 (Hymenoptera, Ichneumonidae, Campopleginae) from Oriental China and Nepal are described (*V. contiguus***sp. nov.** and *V. yunnanensis***sp. nov.** from China; *V. liuae***sp. nov.** and *V. levocarinata***sp. nov.** from Nepal). In addition, two species are reported from China (*V. serpentina* Maheshwary, 1977 and *V. inclyta* (Morley, 1923)) for the first time and all listed species are illustrated. A key to all species from China and Nepal is provided.

## Introduction

The genus *Venturia* Schrottky, 1902 (Ichneumonidae, Campopleginae) is a moderately large genus with 144 valid species worldwide, but predominantly from the Neotropical, Nearctic, and Oriental regions (Gupta and Maheshwary 1977; Wahl 1987; Yu et al. 2016; Vas 2019a, b, 2020; Vas and Di Giovanni 2020). Nine species are known from China and two from Nepal (Yu et al. 2016).

Members of *Venturia* are solitary koinobiont endoparasitoids (Hemerik and Harvey 1999; Jervis et al. 2008; Biddinger et al. 2014). They mainly parasitize the larvae of microlepidoptera that feed in a concealed situation (Wahl 1987), most commonly of the family Pyralidae (He et al. 1996; Eliopoulos et al. 2002; Shaw et al. 2016), and several species were reared indirectly from Vespidae (Sonan 1937) because the caterpillars used for their offspring were parasitized.

Up to now, 36 species of *Venturia* have been recorded from the Oriental area prior to this study (Yu et al. 2016). Two new species are described from southern China, and two species are newly recorded in China in this paper. In addition, two new species are described from Nepal. A key to all species from China and Nepal is provided.

## Material and methods

This study is based on specimens preserved in the Parasitoid Hymenoptera Collection of the Institute of Insect Sciences, Zhejiang University (**ZJUH**) which contains about 0.6 million pinned specimens and about same number of specimens in alcohol collected from all over the China.

The terminology and measurements used follow Broad (2018). All description and measurements were made under ZEISS Stemi 305 microscopes, and all figures were made by digital microscope (VHX-2000C, KEYENCE, Osaka, Japan). Type specimens and other materials are deposited in the Parasitic Hymenoptera Collection of ZJUH.

## Results

### Key to female species of *Venturia* Schrottky from China and Nepal

**Table:** 

1	Face either punctate (sometimes rugose-punctate) or strongly rugose; frons usually rugose, in a few species granulose-punctate	**2**
–	Face granulose or granulose-rugose; frons granulose	**12**
2	Apical half of antenna reddish brown; fore and mid legs from coxae on yellow; hind leg from trochantellus on yellow; mandible and tegula yellow; areolet present, emitting 2m-cu vein a little beyond its middle; anterior transverse carina away from base of propodeum	***V. mongolica* (Kokujev, 1915)**
–	Antenna entirely black or with a white band medially (*V. inclyta*); fore and mid legs from coxae on not wholly yellow; hind leg from trochantellus on not wholly yellow; mandible yellow or blackish brown, tegula yellow or black; areolet present or absent, **if** present, emitting 2m-cu vein a little beyond its middle or its apical part; anterior transverse carina not away from base of propodeum	**3**
3	Mesopleuron with sparse and shallow punctures, and shiny; interocellar distance almost equal to or a little less than ocello-ocular distance; metapleuron shiny	**4**
–	Mesopleuron closely to densely punctate or rugose-punctate, and matte; interocellar distance 1.0–2.5× ocello-ocular distance; metapleuron matte	**5**
4	Fore wing with areolet; malar space matte, 0.6× basal width of mandible; occipital carina beak-like medially; interocellar distance 1.8× distance between median and lateral ocelli	***V. taiwana* (Sonan, 1937)**
–	Fore wing without areolet (Fig. 7A); malar space shiny, 0.2× basal width of mandible (Fig. 7F); occipital carina not beak-like medially; interocellar distance 2.5× distance between median and lateral ocelli (Fig. 7H)	***V. levocarinata* sp. nov.**
5	Lateral longitudinal carina of propodeum absent (Fig. 9D); lateromedian longitudinal carina absent below anterior transverse carina (Fig. 9D)	***V. liuae* sp. nov.**
–	Lateral longitudinal carina of propodeum present; lateromedian longitudinal carina present below anterior transverse carina	**6**
6	Interocellar distance equal to ocello-ocular distance; hind leg entirely black; propodeum long, extending to 0.7 of hind coxa; areolet small with a long stalk	***V. longipropodeum* (Uchida, 1942)**
–	Interocellar distance 1.5–2.5× distance ocello-ocular distance; hind leg usually partly pale, **if** entirely black, then fore wing without areolet; propodeum short to long; areolet absent, or if present then areolet relatively large with a short stalk	**7**
7	Mesopleuron strongly and closely rugose-punctate; mesopleuron, metapleuron and propodeum more or less similar in sculpture	**8**
–	Mesopleuron distinctly punctate, punctures well separated; metapleuron and propodeum punctate to rugose-punctate	**9**
8	Female antenna without a white band; interocellar distance 1.5× ocello-ocular distance (Fig. 14G); metanotum rugose-reticulate; lateromedian longitudinal carina relatively weak below anterior transverse carina (Fig. 14D); face and frons rugose-punctate (Fig. 14F)	***V. yunnanensis* sp. nov.**
–	Female antenna with a white band (Fig. 3); interocellar distance 2.0–2.2× ocello-ocular distance (Fig. 5G); metanotum rugose; lateromedian longitudinal carina strong below anterior transverse carina (Fig. 5D); face rugose and frons punctate (Fig. 5F)	***V. inclyta* (Morley, 1923)**
9	Fore wing with areolet	**10**
–	Fore wing without areolet	**11**
10	Lateromedian longitudinal carina of propodeum weak below anterior transverse carina; area superomedia pentagonal; fore wing areolet not triangular	***V. canescens* (Gravenhorst, 1829)**
–	lateromedian longitudinal carina of propodeum strong below anterior transverse carina; area superomedia triangular; fore wing areolet triangular	***V. himachala* Maheshwary, 1977**
11	Malar space 0.2× basal width of mandible (Fig. 2F); propodeal area basalis not confluent with area superomedia (Fig. 2D); second tergite 2.0× longer than third tergite; 2rs-m vein very close to 2m-cu vein (Fig. 2A)	***V. contiguus* sp. nov.**
–	Malar space 0.5–0.6× basal width of mandible; propodeal area basalis confluent with area superomedia; second tergite a little longer than third tergite; 2rs-m vein not distinctly removed from 2m-cu vein	***V. oditesi* (Sonan, 1939)**
12	Tegula dark brown; trochanters and trochantellus dark brown; mesosoma sculpture superimposed on a granulose surface; hind tibia reddish	***V. roborowskii* (Kokujev, 1915)**
–	Tegula yellow; trochanters and trochantellus not wholly dark brown; mesosoma sculpture superimposed on a granulose surface or not; hind tibia yellow or yellowish brown	**13**
13	Interocellar distance 1.7–2.0× ocello-ocular distance; head not conspicuously swollen and vertex excavate behind; lateromedian longitudinal carina of propodeum angulate and strong below anterior transverse carina	**14**
–	Interocellar distance equal to ocello-ocular distance; head usually swollen and vertex not excavate behind; lateromedian longitudinal carina of propodeum parallel-sided and weak below anterior transverse carina	**15**
14	Area superomedia of propodeum elongated and closed; propodeal carinae strong; apex of propodeum produced and reaching up to 0.7 of hind coxa	***V. hexados* Maheshwary, 1977**
–	Area superomedia of propodeum squarish and open; propodeal carinae weak; propodeum short, not produced apically	***V. quadrata* Maheshwary, 1977**
15	Metasoma smooth and shiny (Fig. 10); petiole rounded and without a distinct postpetiole; thyridium after basal 0.3 of tergite; second tergite 0.7× as long as first tergite	***V. serpentina* Maheshwary, 1977**
–	Metasoma matte; postpetiole swollen and distinctly differentiated from petiole; thyridium in basal 0.3 of tergite; second tergite as long as first tergite	***V. minuta* Maheshwary, 1977**

## Species account

### Subfamily Campopleginae Förster, 1869

#### Genus Venturia

Taxon classificationAnimaliaHymenopteraIchneumonidae

Schrottky, 1902

7C822801-6F2D-561E-98CA-FB3468B03C78

##### Type species.

*Venturia argentina* Schrottky, 1902; by original designation.

##### Diagnosis.

Eye not indented to weakly indented opposite antennal socket; frons with or without a lateromedian longitudinal carina; propodeum long, reaching beyond middle of hind coxa, sometimes extending to apex of hind coxa; area superomedia and area petiolaris usually confluent; lateromedian longitudinal carina close together and more or less parallel to each other; propodeal spiracle round to oval; areolet present to absent; CU&cu-a of hind wing intercepted or not intercepted; claws not pectinate to strongly pectinate; metasoma petiole slender, first metasomal segment round in cross-section of basal 0.3, the suture separating tergite from its sternite lies in the middle or above the middle; ovipositor long, straight to strongly upcurved; male genital paramere weakly to strongly notched apically.

#### Venturia contiguus sp. nov.

Taxon classificationAnimaliaHymenopteraIchneumonidae

CEF05D40-3970-568C-BFA4-C4C34193D12D

http://zoobank.org/111B1B1D-ECF6-4D6C-BA77-4A5853C9DEB5

Figures 1 , 2

##### Materia examined.

***Holotype*:** China • ♀; Fujian, Nanping; 21.IX.2002; Xiao-Xia Yu leg.; No. 20025513. ***Paratype***: China • 1♀; Zhejiang, Songyang; 7.VII.1982; Han-Lin Chen leg.; No. 924532.

##### Comparative diagnosis.

In the key by Gupta and Maheshwary (1977), this species keys out as *V. oditesi* (Sonan, 1939) from China and Myanmar because of the missing areolet on the fore wing. It can be easily distinguished from *V. oditesi* by the following: malar space approximately 0.2× basal width of mandible, propodeal area basalis not confluent with area superomedia, second tergite 2.0× longer than third tergite, 2rs-m vein very close to 2m-cu vein, hind femur blackish brown, hind tibia blackish brown medially, and metasoma wholly blackish with apical segments blackish brown.

This species is also similar to *V. oblongata* Gupta & Maheshwary, 1977 from Singapore, but differs from it by having the following: face rugulose-punctate, malar space 0.2× basal width of mandible, pronotum rugose-punctate dorsally, mesoscutum punctate and punctate-reticulate apically, propodeal area lateralis rugose-punctate, area basalis not confluent with area superomedia, area external punctate, hind femur blackish brown, and metasoma wholly blackish with apical segments blackish brown.

##### Description.

**Figure 1.**
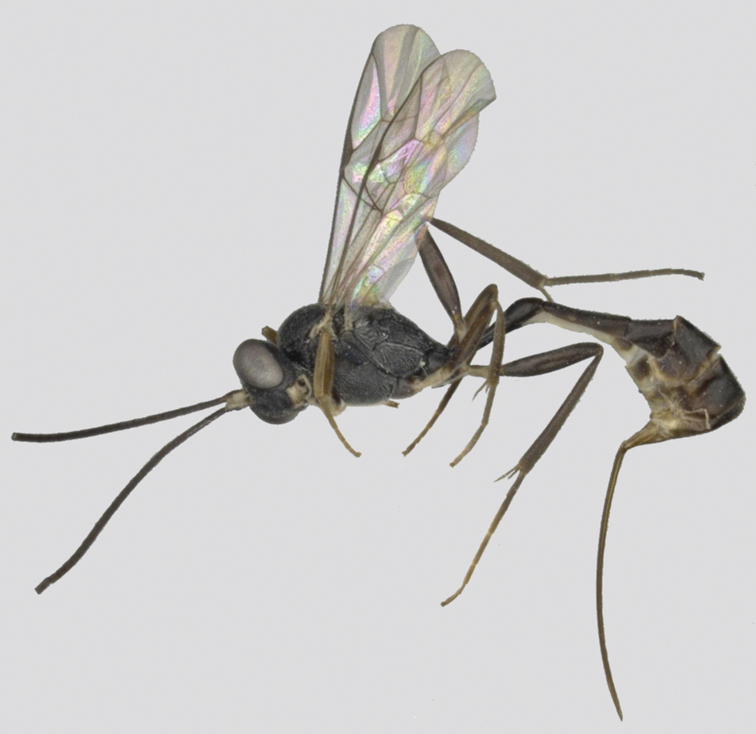
*Venturia contiguus* sp. nov., female, habitus, lateral view.

**Female** holotype (Fig. 1). Body length 7.2 mm, fore wing length 3.9 mm.

**Figure 2.**
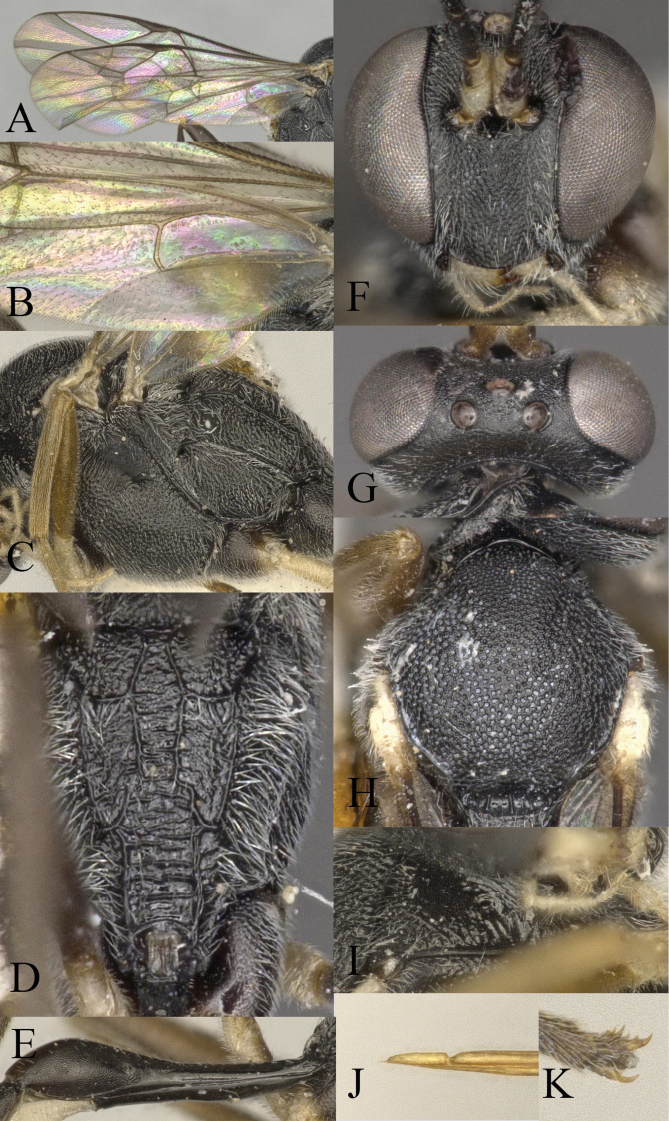
*Venturia contiguus* sp. nov., female **A** fore wing **B** hind wing **C** mesosoma, lateral view **D** propodeum, dorsal view **E** first metasomal segment, lateral view **F** head, anterior view **G** head, dorsal view **H** mesoscutum, dorsal view **I** pronotum, lateral view **J** tip of ovipositor, lateral view **K** hind tarsal claw.

***Head*.** Antenna with 37 flagellomeres; first flagellomere 1.2× longer than second flagellomere. Face (Fig. 2F) rugulose-punctate, somewhat less pronounced laterally. Clypeus matte, weakly punctate. Malar space finely granulate, 0.2× basal width of mandible. Mandible with lamella more prominent in the basal 0.5. Frons granulate-punctate, median carina indistinct. Vertex granulate. Interocellar distance (Fig. 2G) 2.1× ocello-ocular distance and 2.0× distance between median and lateral ocelli. Temple subpolished, ca 0.4× length of the eye in dorsal view. Occipital carina evenly arched, joining hypostomal carina at mandible base.

***Mesosoma*.** Pronotum (Fig. 2I) rugose-punctate dorsally, trans-striate laterally. Mesoscutum (Fig. 2H) punctate, rugulose-punctate in notaulic area, punctate-reticulate apically. Scutellum punctate anteriorly, rugose-punctate posteriorly. Metanotum rugose-punctate. Mesopleuron (Fig. 2C) punctate, trans-striate below tegula, speculum smooth. Propodeum (Fig. 2D) with area basalis trapezoid; area superomedia long and narrow with rugosity, not confluent with area basalis but confluent with area petiolaris; area petiolaris trans-striate; area external punctate; area dentipara rugose-punctate; area lateralis rugose-punctate; lateromedian longitudinal carina almost parallel; propodeal spiracle small, oval. Propodeum extending to 0.7 of hind coxa.

***Wing*.** Fore wing (Fig. 2A) areolet absent. 2rs-m slightly in front of the 2m-cu by only 0.2× of its length. RS ca 1.6× longer than 2r&RS. 1cu-a opposite M&RS, inclivous. External angles of second discal cell acute (70°). Hind wing (Fig. 2B) with CU&cu-a intercepted at lower 0.3 of its length. Distal abscissa of CU connected to CU&cu-a, spectral.

***Legs*.** Hind femur 5.3× longer than wide. Inner spur ca 0.6× as long as first tarsomere of hind tarsus. Tarsal claws pectinate (Fig. 2K).

***Metasoma*.** Apical tergites from third on compressed. First segment (Fig. 2E) long and slender, ca 3.1× longer than its apical width, without glymma; dorsolateral carina of first tergite missing; petiole ca 6.0× width; suture separating first tergite from sternite situated mid height at basal third of first metasomal segment. Second tergite finely granulate, relatively long and slender, 0.8× first tergite, 2.0× third tergite, 3.4× its apical width; thyridium oval, located at basal 0.4 length of second tergite. Posterior margins of sixth and seventh tergites medially concave. Ovipositor sheath ca 1.5× longer than hind femur, ovipositor ca 2.5× longer than hind femur. Ovipositor upcurved apically, dorsal preapical notch strong, tip acute (Fig. 2J).

***Colour*.** Black. Mandible except teeth, scape and pedicel except laterally, palpi, tegula, fore and mid coxae in apical half and all trochanters, yellow. Coxae ventrally blackish brown. Rest of fore and mid legs yellowish brown to blackish brown, with apical tarsal segment blackish brown. Hind coxa in apical half and trochanter, yellow. Remainder of hind leg blackish brown with tibia basally and apically darker. Metasoma wholly blackish with apical segments blackish brown.

##### Distribution.

China (Fujian, Zhejiang).

##### Etymology.

Name derived from “contiguus” (Latin for “near”), because 2rs-m vein situated close to 2m-cu vein.

#### Venturia inclyta

Taxon classificationAnimaliaHymenopteraIchneumonidae

(Morley, 1923)

FC3027D5-FC5C-5B5C-A618-CF8AA349406D

Figures 3 , 4 , 5

Cymodusa inclyta Morley, 1923: 8; Townes and Gupta 1961: 234.
Venturia inclyta Gupta and Maheshwary 1977: 93–95.

##### Materia examined.

China • 1♀; Fujian, Shaowu; 5.XII.1981; Jian-Hong Qiu leg.; No. 816433 • 5♀4♂; Guangxi, Lingchuan; 1983; Gui-Yu Li leg.; No. 835369(9) • 1♀1♂; Guangxi, Nanning; 17.III.1986; Wei-Bao Huang leg.; No. 860822.

**Figure 3.**
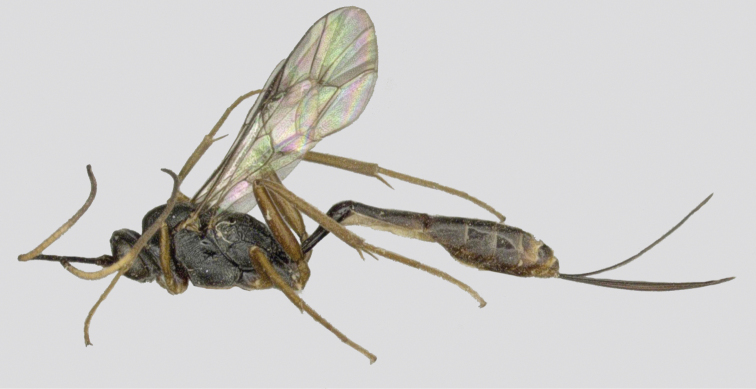
*Venturia inclyta*, female, habitus, lateral view.

##### Male

**Figure 4.**
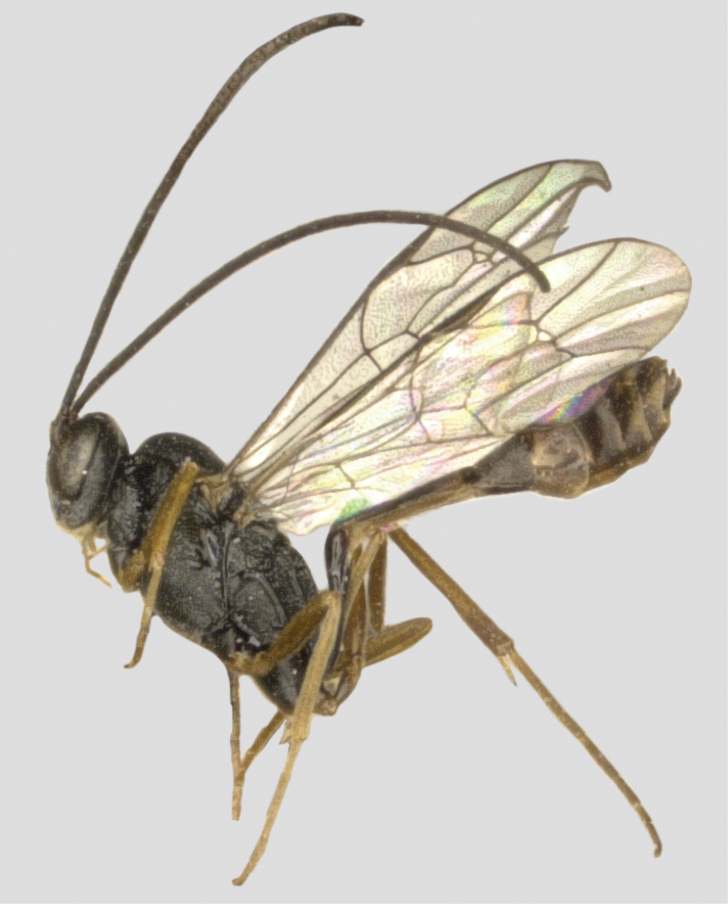
*Venturia inclyta*, male, habitus, lateral view.

**(Fig. 4).** Antenna without a white band, face rugose-punctate, propodeal carinae strong, metasoma strongly compressed apically, otherwise similar to female.

##### Variation.

Antenna with 41–43 flagellomeres, first flagellomere 1.4–1.6× second flagellomere, malar space 0.3–0.5× basal width of mandible, ovipositor 2.4–2.8× the length of hind femur, ovipositor sheath 2.1–2.6× the length of hind femur.

##### Distribution.

China (Fujian, Guangxi), India, Nepal, Sri Lanka. New record for China.

**Figure 5.**
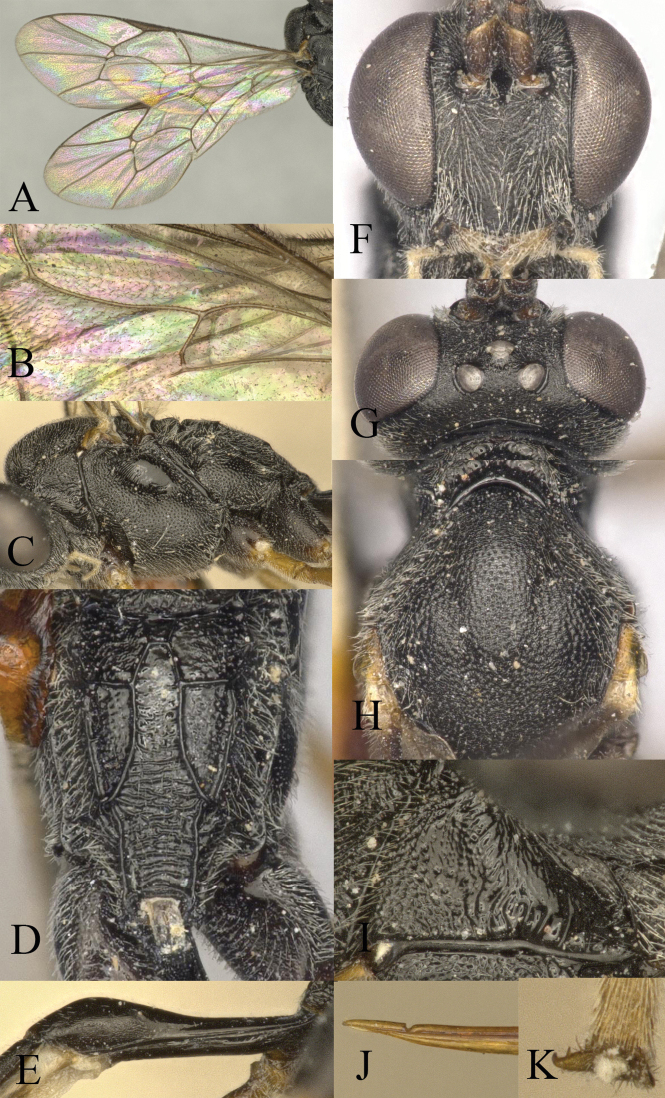
*Venturia inclyta*, female **A** fore wing **B** hind wing **C** mesosoma, lateral view **D** propodeum, dorsal view **E** first metasomal segment **F** head, anterior view **G** head, dorsal view **H** mesoscutum, dorsal view **I** pronotum, lateral view **J** tip of ovipositor, lateral view **K** hind tarsal claw.

#### Venturia levocarinata sp. nov.

Taxon classificationAnimaliaHymenopteraIchneumonidae

19D7A867-6709-595D-8572-9E7EE8EDB063

http://zoobank.org/4FAC75BD-5F83-4DE7-B391-50D7C7577CD0

Figures 6 , 7

##### Materia examined.

***Holotype*:** Nepal • ♀; Nepal, Tansen; 12.VII.2014; Bin-Bin Xu leg.; No. 201502137.

##### Comparative diagnosis.

In the key by Gupta and Maheshwary (1977), this species keys out to *V. inquinata* (Morley, 1913) from India, but differs from *V. inquinata* by the following: face rugose-punctate, teeth with an elevated carina, malar space 0.2× basal width of mandible, mandible without lamella, mandible blackish brown, and postpetiole reddish brown.

This species is also similar to *V. taiwana* (Sonan, 1937) from Taiwan province of China, but differs from it by the following: clypeus without a median apical tooth, teeth with an elevated carina, malar space smooth and shiny, 0.2× basal width of mandible, temple not strongly swollen, fore wing without areolet, tegula black, mid trochanter and femur except apex blackish, hind femur black, and metasoma from third tergite on lateral surface reddish brown with a black dorsal stripe.

This species is similar to *V. prolixa* Wahl, 1987 from America, but differs from latter by having teeth with an elevated carina, propodeal median area not granulate, area external rugose-punctate, area dentipara rugose-reticulate and not on a granulate surface, hind femur ca 11.0× longer than wide, tegula black, hind leg black except extreme base of first tarsomere yellowish brown, and metasoma with second tergite black, laterally brownish, from third tergite on lateral surface reddish brown with a black dorsal stripe.

##### Description.

**Figure 6.**
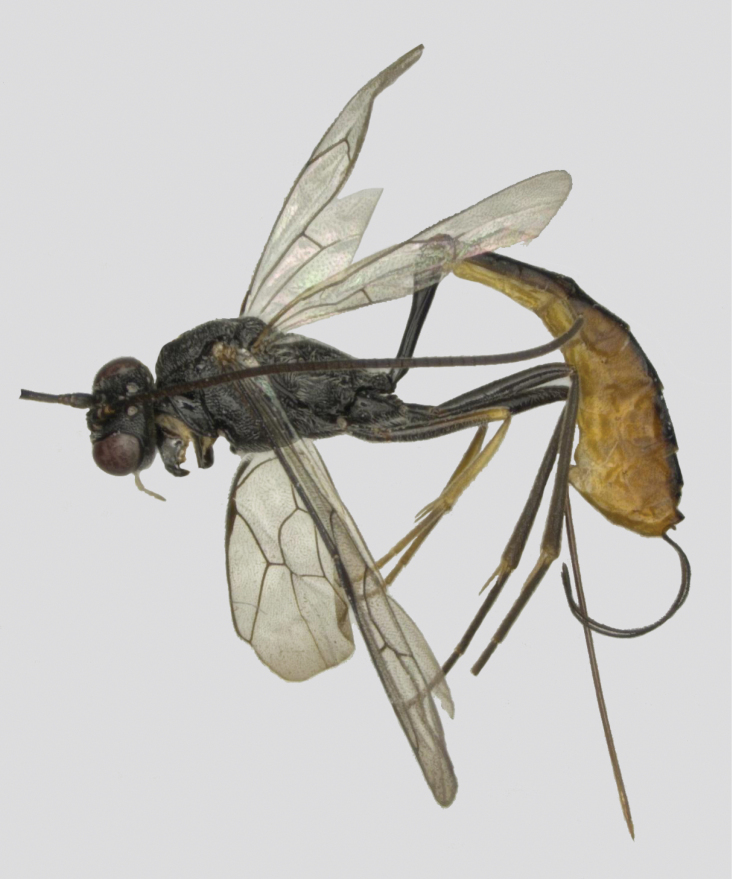
*Venturia levocarinata* sp. nov., female, habitus, lateral view.

**Female** holotype (Fig. 6). Body length 15.0 mm, fore wing length 9.0 mm.

**Figure 7.**
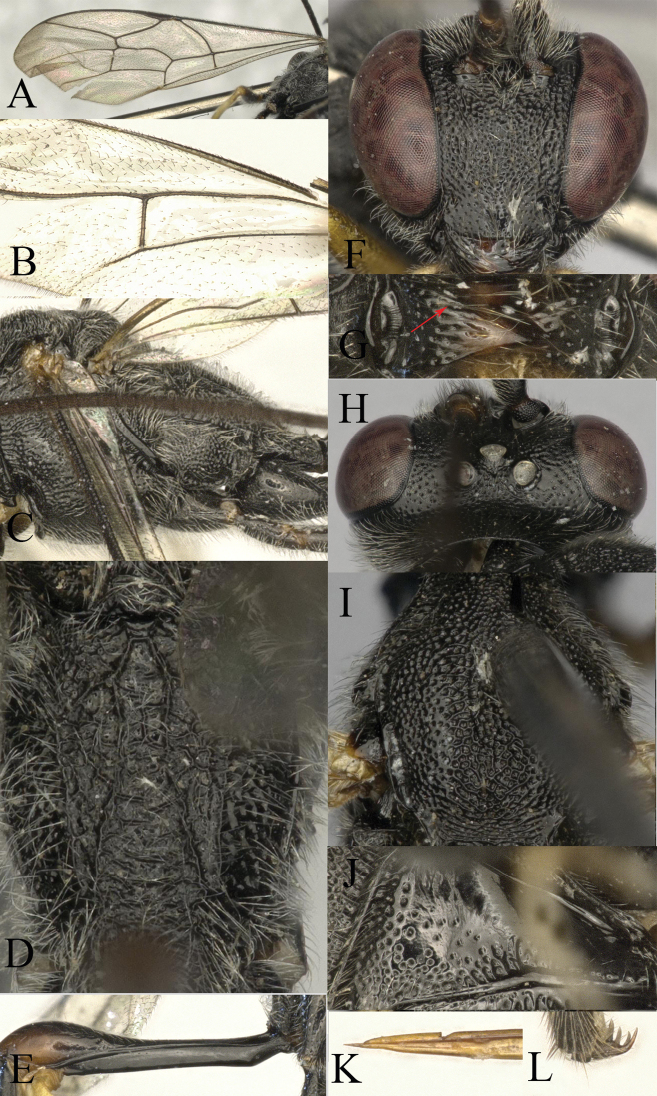
*Venturia levocarinata* sp. nov., female **A** fore wing **B** hind wing **C** mesosoma, lateral view **D** propodeum, dorsal view **E** first metasomal segment **F** head, anterior view **G** mandible, anterior view **H** head, dorsal view **I** mesoscutum, dorsal view **J** pronotum, lateral view **K** tip of ovipositor, lateral view **L** hind tarsal claw.

***Head*.** Antenna at least with 48 flagellomeres (apex missing); first flagellomere 1.4× longer than second flagellomere. Face (Fig. 7F) rugose-punctate, laterally more superficial. Clypeus smooth and shiny, punctate. Malar space smooth and shiny, partly granulate, 0.2× basal width of mandible. Mandible (Fig. 7G) without lamella, with an elevated carina on outer surface. Frons rugose-punctate, median carina distinct. Vertex shallowly to deeply punctate. Ocellar region small. Interocellar distance (Fig. 7H) 0.9× ocello-ocular distance and 2.5× distance between median and lateral ocelli. Temple subpolished, densely punctate below, ca 0.5× length of the eye. Occipital carina evenly arched, joining hypostomal carina far before mandible base.

***Mesosoma*.** Pronotum (Fig. 7J) punctate dorsally, smooth and shiny medially, short striate laterally. Mesoscutum (Fig. 7I) punctate, rugose in notaulic area, rugose-reticulate apically. Scutellum punctate anteriorly, rugose-punctate posteriorly. Metanotum rugose-reticulate. Mesopleuron (Fig. 7C) rugose-punctate above, punctate below, weakly trans-striate below tegula; speculum smooth and shiny. Metapleuron rugose-punctate above, punctate below. Propodeum (Fig. 7D) with area basalis trapezoid; area superomedia small and narrow, confluent with area petiolaris, trans-striate; area external rugose-punctate; area dentipara rugose-reticulate; area lateralis rugose-punctate; all carinae distinct; propodeal spiracle oval. Propodeum extending to 0.9 of hind coxa.

***Wing*.** Fore wing (Fig. 7A) without areolet, and distance between 2rs-m and 2m-cu ca 0.6× length of 2rs-m. RS ca 1.4× longer than 2r&RS. 1cu-a opposite M&RS. External angles of second discal cell acute (75°). Hind wing (Fig. 7B) with CU&cu-a slightly inclivous and not intercepted. Distal abscissa of CU not connected to CU&cu-a.

***Legs*.** Coxae weakly punctate. Hind femur ca 11.0× longer than wide. Inner spur ca. 0.5 as long as first tarsomere of hind tarsus. Tarsal claws strongly pectinate (Fig. 7L).

***Metasoma*.** Apical tergites from third on slightly compressed. First segment (Fig. 7E) long and slender, ca 6.3× longer than apical width, without glymma; dorsolateral carina of first tergite missing; petiole ca 6.5× longer than wide. Suture separating first tergite from sternite situated above the mid-height at basal third of first metasomal segment. Second tergite finely granulate, long and slender, 0.9× first tergite, 1.8× third tergite, 5.0× its apical width; thyridium oval, located at basal 0.4 length of second tergite. Third tergite 2.8× longer than its apical width. Posterior margins of sixth and seventh tergites medially concave. Ovipositor sheath ca 1.8× longer than hind femur, ovipositor ca 2.4× longer than hind femur. Ovipositor nearly straight, dorsal preapical notch strong, tip acute (Fig. 7K).

***Colour*.** Mandible blackish brown, subapically brownish. Tegula black. All coxae black. Fore legs missing. Mid trochanter and femur except apex blackish, remainder of leg yellowish brown. Hind leg black except extreme base of first tarsomere yellowish brown. Petiole black and postpetiole reddish brown, second tergite black, laterally brownish, from third tergite on lateral surface reddish brown with a black dorsal stripe.

##### Distribution.

Nepal.

##### Etymology.

Name derived from “levo” (Latin for “raised”) and “carinata” (Latin for “carina”), because teeth with elevated carina on outer surface.

#### Venturia liuae sp. nov.

Taxon classificationAnimaliaHymenopteraIchneumonidae

B6439B79-723F-5296-9DBC-B44DCF6C23EB

http://zoobank.org/7E2F1CB3-B112-4A32-B2EE-117B10E979D5

Figures 8 , 9

##### Materia examined.

***Holotype*:** Nepal • ♀; Nepal, Kathmandu Nagarkot; 24.VII.2013; Zhen Liu leg.; No. 201406299.

##### Comparative diagnosis.

In the key by Gupta and Maheshwary (1977) this species keys out as *V. ahlensis* Maheshwary, 1977 from India, because the propodeal lateromedian longitudinal carina and lateral longitudinal carina are absent, but it can be easily distinguished from *V. ahlensis* by the following: areolet small with a long stalk, malar space ca 0.45× basal width of mandible, and area superomedia region rugulose.

This species is also similar to *V. himachala* Gupta & Maheshwary, 1977 from Indian and Nepal, but differs from latter by the following: frons rugulose with median carina absent, interocellar distance 1.3× ocello-ocular distance, anterior part of median lobe of mesoscutum with indistinct punctures, and propodeal lateromedian carina absent below anterior transverse carina.

##### Description.

**Figure 8.**
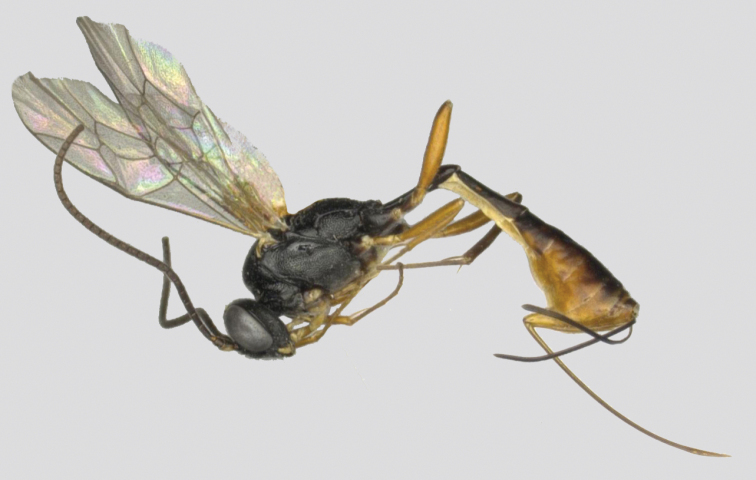
*Venturia liuae* sp. nov., female, habitus, lateral view.

**Female** holotype (Fig. 8). Body length 6.5 mm, fore wing length 4.0 mm.

**Figure 9.**
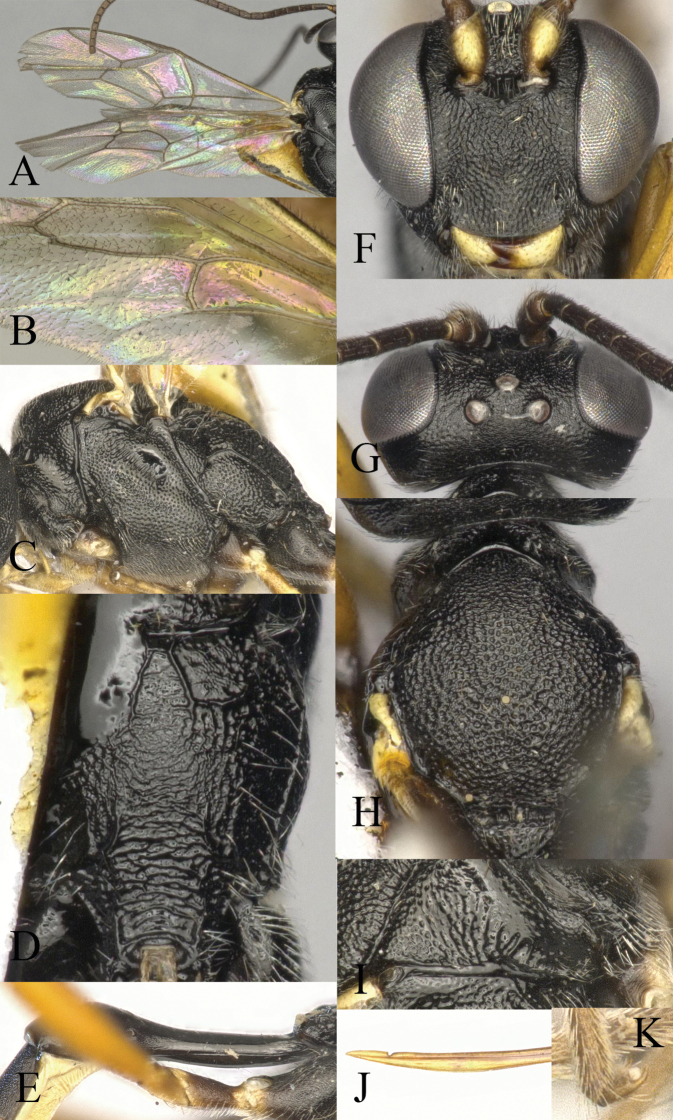
*Venturia liuae* sp. nov., female **A** fore wing **B** hind wing **C** mesosoma, lateral view **D** propodeum, dorsal view **E** fisrt metasomal segment, lateral view **F** head, anterior view **G** head, dorsal view **H** mesoscutum, dorsal view **I** pronotum, lateral view **J** tip of ovipositor, lateral view **K** hind tarsal claw.

***Head*.** Antenna with at least 33 flagellomeres (apex missing), length of first flagellomere ca 1.3× longer than second flagellomere; face (Fig. 9F) rugose-punctate, punctures stronger and coalescent centrally, and shallow laterally; malar space granulate, ca 0.45× basal width of mandible; mandible with a weak lamella; frons rugulose, median carina absent; vertex rugulose-punctate; temple shallowly punctate, ca 0.5× length of the eye; ocellar region punctate; interocellar distance (Fig. 9G) 1.3× ocello-ocular distance and 2.0× distance between median and lateral ocelli; occipital carina evenly arched, joining hypostomal carina before mandible base.

***Mesosoma*.** Pronotum (Fig. 9I) trans-striate laterally, closely punctate dorsally; mesoscutum (Fig. 9H) punctate, matte, anterior part of median lobe with indistinct punctures; scutellum punctate; metanotum rugose-punctate; mesopleuron (Fig. 9C) densely punctate, punctures separated by less than their diameter, weakly striate below subtegular ridge; metapleuron similar to mesopleuron except that the punctures little denser. Propodeum (Fig. 9D) with area superomedia rugulose, area petiolaris rugose-striate; lateral longitudinal carina absent; lateromedian longitudinal carina absent below anterior transverse carina; propodeal spiracle oval; propodeum projecting at 0.5 of hind coxa.

***Wing*.** Fore wing (Fig. 9A) areolet small with a long stalk, the height of areolet ca 0.7× as long as stalk, emitting 2m-cu vein from its apical part. RS ca 1.8× longer than 2r&RS. 1cu-a opposite M&RS. External angles of second discal cell acute (70°). Hind wing (Fig. 9B) with CU&cu-a intercepted at lower 0.35 of its length.

***Legs*.** Hind femur 5.0× longer than wide. Inner spur of hind tibia ca 0.45× as long as first tarsomere of hind tarsus. Tarsal claws pectinate (Fig. 9K).

***Metasoma*.** Apical tergites from third on slightly compressed. First segment (Fig. 9E) long and slender, ca 3.8× longer than its apical width, without glymma; dorsolateral carina of first tergite missing; petiole ca 5.0× longer than high. Suture separating first tergite from sternite situated mid-height at basal third of first metasomal segment. Second tergite granulate, long and slender, 0.9× first tergite, 2.6× its apical width; thyridium oval, small, its distance from basal margin of tergite ca 3.0× its length. Third tergite 1.3× longer than its apical width. Posterior margins of sixth and seventh tergites medially concave. Ovipositor sheath ca 1.8× longer than hind femur, ovipositor ca 3.0× longer than hind femur, ovipositor gradually upcurved, dorsal preapical notch strong, tip acute (Fig. 9J).

***Colour*.** Black. Scape narrowly in front, mandible except teeth, palpi, tegula, extreme apices of fore and mid coxae, fore trochanter and trochantellus of mid trochanter, yellow; remainder of fore leg yellowish brown with apical segment dark brown and remainder of mid leg yellowish brown with tarsus blackish brown; hind leg with trochanter blackish brown, trochantellus yellowish brown with externally more brownish, femur yellowish brown but apically blackish, tibia brownish with base and apex blackish, tarsus blackish brown; metasoma with first and second segment wholly black, dorsal surface from third segment on black but laterally reddish brown.

##### Distribution.

Nepal.

##### Etymology.

This species is named in honor of Dr Zhen Liu, the collector of the holotype.

#### Venturia serpentina

Taxon classificationAnimaliaHymenopteraIchneumonidae

Maheshwary, 1977

0B6507EF-7C00-569C-AD1D-CEEC1453DF91

**Figure 10.**
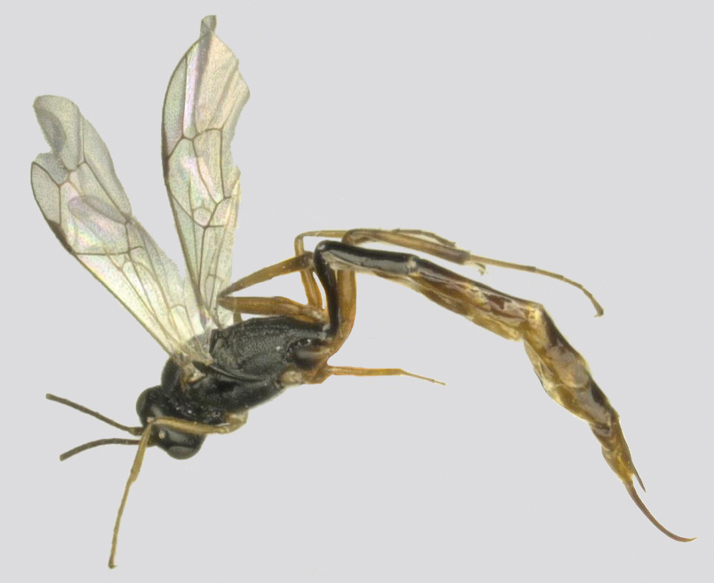
*Venturia serpentina*, female, habitus, lateral view.

**Figure 11.**
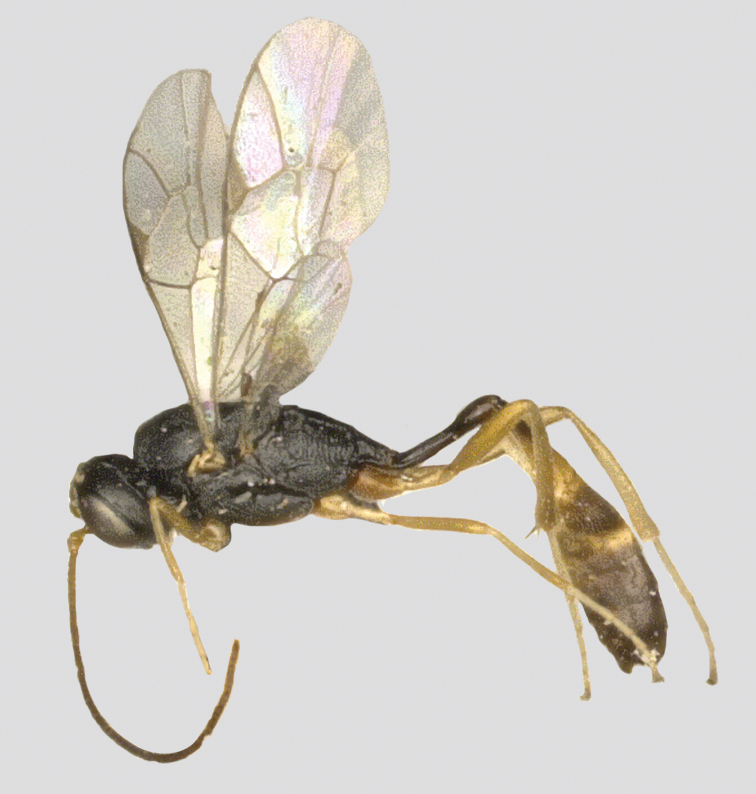
*Venturia serpentina*, male, habitus, lateral view.

**Figure 12.**
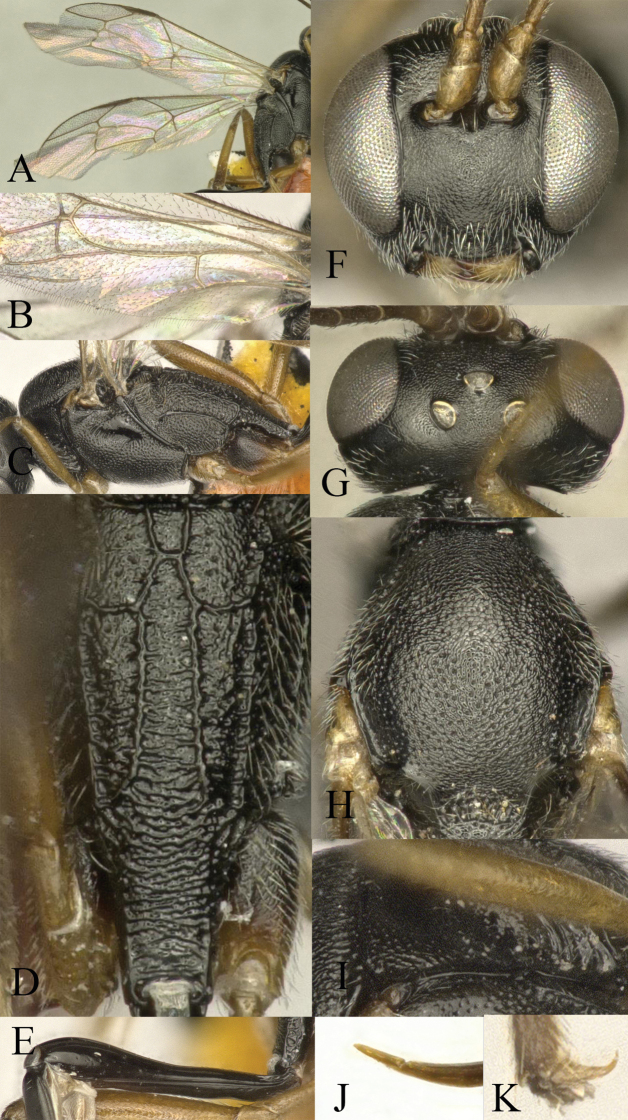
*Venturia serpentina*, female **A** fore wing **B** hind wing **C** mesosoma, lateral view **D** propodeum, dorsal view **E** first metasomal segment **F** head, anterior view **G** head, dorsal view **H** mesoscutum, dorsal view **I** pronotum, lateral view **J** tip of ovipositor, lateral view **K** hind tarsal claw.

Figures 10 , 11 , 12

Venturia serpentina Maheshwary in Gupta and Maheshwary 1977: 114–115.

##### Materia examined.

China • 1♀; Guangdong, Shixing; 25.V.2002; Zai-Fu Xu leg.; No. 201806114 • 1♂; Zhejiang, Hangzhou; 8.VIII.1981; Jun-Hua He leg.; No. 815229.

##### Male

**(Fig. 11).** Interocellar distance 1.7× ocello-ocular distance and 2.5× distance between median and lateral ocelli. Fore wing 1cu-a distad of M&RS by 0.3 its length. Metasoma not snake-like. Hind coxa yellow, hind tibia wholly yellow.

##### Distribution.

China (Guangdong, Zhejiang), Myanmar. New record for China.

#### Venturia yunnanensis sp. nov.

Taxon classificationAnimaliaHymenopteraIchneumonidae

4C17214B-4F24-53F4-9E58-E02D8865F6CF

http://zoobank.org/31AF3FED-3F01-433A-BB4E-83D5E11DDD2C

Figures 13 , 14

##### Materia examined.

***Holotype*:** China • ♀; Yunnan, Xishuangbanna; 20.VI.2018; 21°44.75'N, 100°26.00'E; 1610 m; Malaise trap; No. 20180823.

##### Comparative diagnosis.

In the key by Gupta and Maheshwary (1977), this species keys out to *V. tectonae* (Perkins, 1936) from Myanmar, but differs from it by the following: interocellar distance 1.2× ocello-ocular distance, second tergite 1.5× third tergite, malar space weakly punctate, tegula yellowish brown, and differently coloured metasoma.

This species is also similar to *V. anchisteus* Wahl, 1987 from Mexico, but differs from it by the following: propodeal area external punctate, area dentipara rugose-reticulate, the height of areolet ca equal to the length of stalk, second metasomal tergite ca 1.7× its apical width, and hind tibia except base and apex yellowish brown.

##### Description.

**Figure 13.**
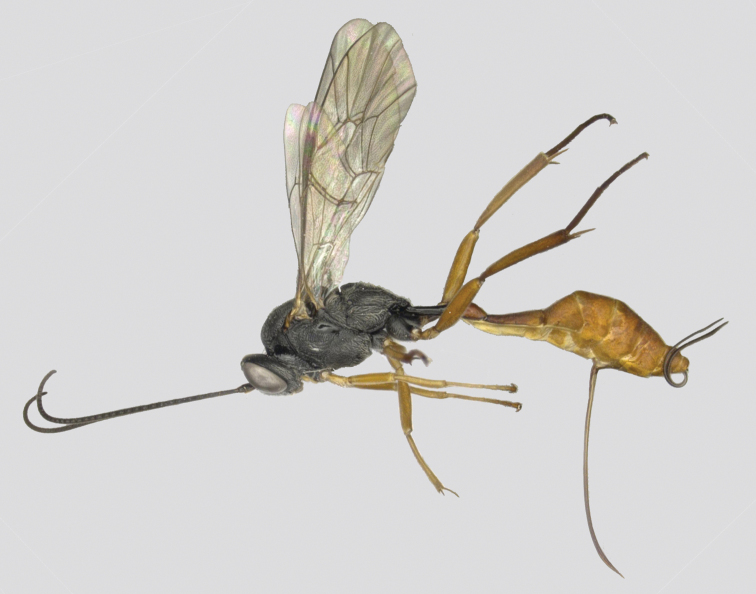
*Venturia yunnanensis* sp. nov., female, habitus, lateral view.

**Female** holotype (Fig. 13). Body length 13.0 mm, fore wing length 8.8 mm.

**Figure 14.**
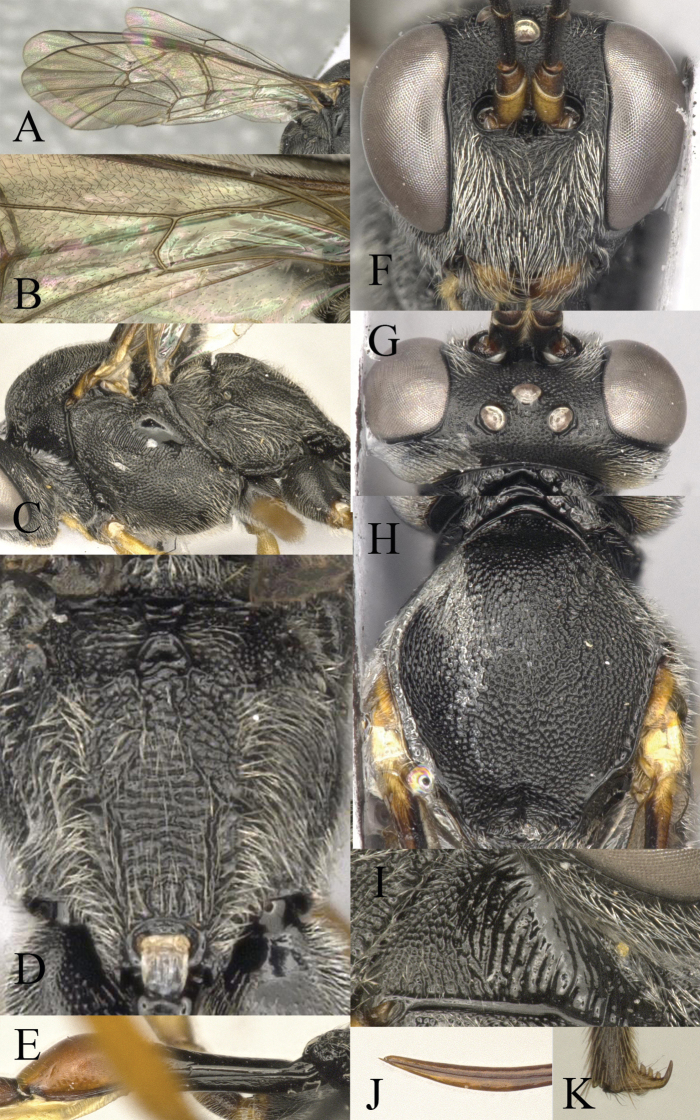
*Venturia yunnanensis* sp. nov., female **A** fore wing **B** hind wing **C** mesosoma, lateral view **D** propodeum, dorsal view **E** first metasomal segment, lateral view **F** head, anterior view **G** head, dorsal view **H** mesoscutum, dorsal view **I** pronotum, lateral view **J** tip of ovipositor, lateral view **K** hind tarsal claw.

***Head*.** Antenna a little shorter than fore wing, with 42 flagellomeres; first flagellomere ca 1.5× longer than second flagellomere. Face (Fig. 14F) densely rugose-punctate. Clypeus punctate, punctures sparser than on face. Malar space weakly punctate, 0.35× basal width of mandible. Frons rugose-punctate, punctate on sides; with median carina. Vertex matte, shallowly punctate. Ocellar region punctate. Interocellar distance (Fig. 14G) 1.5× ocello-ocular distance and 2.4× distance between median and lateral ocelli. Temple shallowly punctate, ca 0.4× length of the eye. Occipital carina evenly arched, joining hypostomal carina at mandible base.

***Mesosoma*.** Pronotum (Fig. 14I) rugose-punctate dorsally, trans-striate laterally. Mesoscutum (Fig. 14H) punctate, rugose in notaulic region. Scutellum punctate anteriorly, rugose-punctate posteriorly. Metanotum rugose-reticulate. Mesopleuron (Fig. 14C) rugose-punctate above, punctate below, trans-striate below tegula. Metapleuron rugose-punctate above, rugose-reticulate below. Propodeum (Fig. 14D) with area basalis trapezoid; area superomedia region small, rugose and confluent with area petiolaris; area petiolaris trans-striate; area external punctate; area dentipara rugose-reticulate; area lateralis rugose-punctate; lateromedian longitudinal carina relatively weak below anterior transverse carina, and narrow posteriorly; propodeal spiracle oval. Propodeum extending to 0.5 of hind coxa.

***Wing*.** Fore wing (Fig. 14A) with relatively small, petiolate areolet, height of areolet ca equal to the length of stalk, emitting 2m-cu vein from its apical part. RS ca 1.8× longer than 2r&RS. 1cu-a opposite M&RS. External angles of second discal cell acute (75°). Hind wing (Fig. 14B) with CU&cu-a intercepted at lower 0.35× of its length. Distal abscissa of CU connected to CU&cu-a, spectral.

***Legs*.** Coxae weakly punctate. Hind femur ca 5.3× longer than wide. Inner spur ca 0.45× first tarsomere of hind tarsus. Tarsal claws pectinate (Fig. 14K).

***Metasoma*.** Apical tergites from third on slightly compressed. First segment (Fig. 14E) long and slender, ca 3.9× longer than its apical width, without glymma; dorsolateral carina of first tergite missing; petiole ca 5.5× longer than wide. Suture separating first tergite from sternite situated mid-height at basal third of first metasomal segment. Second tergite finely granulate, relatively long and slender, 0.7× first tergite, 1.5× third tergite, 1.7× its apical width; thyridium oval, its distance from basal margin of tergite ca 2.5× its length. Third tergite 1.15× longer than its apical width. Posterior margins of sixth and seventh tergites medially concave. Ovipositor sheath ca 1.8× longer than hind femur, ovipositor ca 2.6× longer than hind femur. Ovipositor upcurved apically, dorsal preapical notch absent, tip acute (Fig. 14J).

***Colour*.** Mandible except teeth, palpi, tegula, scape and pedicel in front, fore and middle legs from the apices of coxae onward, yellowish brown, femora and tarsus darker. Hind leg with tarsus, base and apex of tibia, base of trochanter, blackish brown; remainder of the hind leg yellowish brown. First metasomal segment black with postpetiole reddish brown, second segment reddish brown with apically lighter, remainder of the tergites light reddish orange.

##### Distribution.

China (Yunnan).

##### Etymology.

Name derived from the name of type locality of species.

## Supplementary Material

XML Treatment for Venturia

XML Treatment for Venturia contiguus

XML Treatment for Venturia inclyta

XML Treatment for Venturia levocarinata

XML Treatment for Venturia liuae

XML Treatment for Venturia serpentina

XML Treatment for Venturia yunnanensis

## References

[B1] Biddinger DJ Leslie TW (2014) Observation on the biological control agents of the American plum borer (Lepidoptera: Pyralidae) in Michigan cherry and plum orchards. Great Lakes Entomologist 47(1–2): 51–65.

[B2] Broad GR Shaw MR Fitton MG (2018) Ichneumonid wasps (Hymenoptera: Ichneumonidae): their classification and biology. Handbooks for the Identification of British Insects 7(12): 1–418.

[B3] Eliopoulos PA Athanasiou CG Buchelos CH (2002) Occurrence of hymenopterous parasitoids of stored product pests in Greece. Bulletin Olib Srop 25(3): 127–139.

[B4] Gupta VK Maheshwary S (1977) Ichneumonologia Orientalis, Part IV. The tribe Porizontini (=Campoplegini) (Hymenoptera: Ichneumonidae). Oriental Insects Monograph 5: 1–267.

[B5] He JH Chen XX Ma Y (1996) Hymenoptera: Ichneumonidae. Economic Insect Fauna of China, Science Press, Beijing, 697 pp. [in Chinese]

[B6] Hemerik L Harvey JA (1999) Flexible larval development and the timing of destructive feeding by a solitary endoparasitoid: an optimal foraging problem in evolutionary perspective. Ecological Entomology 24(3): 308–315. 10.1046/j.1365-2311.1999.00203.x

[B7] Jervis MA Ellers J Harvey JA (2008) Resource acquisition, allocation, and utilization in parasitoid reproductive strategies. Annual Review of Entomology 53: 361–385. 10.1146/annurev.ento.53.103106.093433 17877453

[B8] Shaw MR Horstmann K Whiffin AL (2016) Two hundred and twenty-five species of reared western Palaearctic Campopleginae (Hymenoptera: Ichneumonidae) in the National Museums of Scotland, with descriptions of new species of *Campoplex* and *Diadegma*, and records of fifty-five species new to Britain. Entomologist’s Gazette 67: 177–222.

[B9] Sonan J (1937) Two new species and one new genus of Hymenoptera. Transactions of the Natural History Society of Formosa 27(166): 169–174.

[B10] Vas Z (2019a) Contributions to the taxonomy, identification, and biogeography of *Casinaria* Holmgren and *Venturia* Schrottky (Hymenoptera: Ichneumonidae: Campopleginae). Zootaxa 4664(3): 351–364. 10.11646/zootaxa.4664.3.3 31716665

[B11] Vas Z (2019b) New species and new records of Campopleginae from the Palaearctic region (Hymenoptera: Ichneumonidae). Folia Entomologica Hungarica 80: 247–271. 10.17112/FoliaEntHung.2019.80.247

[B12] Vas Z (2020) New species and records of Afrotropical, Oriental and Palaearctic *Venturia* Schrottky, 1902 (Hymenoptera: Ichneumonidae: Campopleginae). Opuscula Zoologica Instituti Zoosystematici et Oecologici Universitatis Budapestinensis 51(2): 97–114. 10.18348/opzool.2020.2.97

[B13] Vas Z Di Giovanni F (2020) New species and records of Afrotropical Campopleginae (Hymenoptera: Ichneumonidae). Folia Entomologica Hungarica 81: 105–114.

[B14] Wahl DB (1987) A revision of *Venturia* north of Central America (Hymenoptera: Ichneumonidae). The University of Kansas Science Bulletin 53(6): 275–356.

[B15] Yu DS van Achterberg C Horstmann K (2016) World Ichneumonoidea 2015. Taxonomy, Biology, Morphology and Distribution. Flash drive. Taxapad, Vancouver.

